# Protocol to extract and measure ubiquinone and rhodoquinone in murine tissues

**DOI:** 10.1016/j.xpro.2025.103880

**Published:** 2025-06-12

**Authors:** Madison Jerome, Jessica B. Spinelli

**Affiliations:** 1 Program in Molecular Medicine, University of Massachusetts Chan Medical School, Worcester, MA 01605, USA

**Keywords:** cell biology, metabolism, metabolomics

## Abstract

Ubiquinone (UQ) and rhodoquinone (RQ) are electron carriers for the electron transport chain (ETC). Here, we present a protocol for measuring UQ and RQ in mitochondria purified from murine tissues. We describe steps for isolating mitochondria by centrifugation, isolating UQ and RQ by biphasic extraction, and normalizing samples to the protein content of the mitochondrial pellet. We then detail procedures for analyzing UQ and RQ by integrating peak areas for UQ-9 and RQ-9 (abundant in mice) or UQ-10 and RQ-10 (abundant in human). Thus, through enrichment of mitochondria, we establish a method to measure UQ and RQ in tissues.

For complete details on the use and execution of this protocol, please refer to Valeros et al.^1^

## Highlights

• A method to detect rhodoquinone in mammalian tissues by LC-MS
• Details on mitochondrial purification from tissues for rhodoquinone detection
• Biphasic metabolite extraction protocol to enrich for quinones
• Protein quantification of extracted material to equalize LC-MS input

## Abstract (editor highlights)

Publisher’s note: Undertaking any experimental protocol requires adherence to local institutional guidelines for laboratory safety and ethics.

## Graphical abstract

**Figure:**
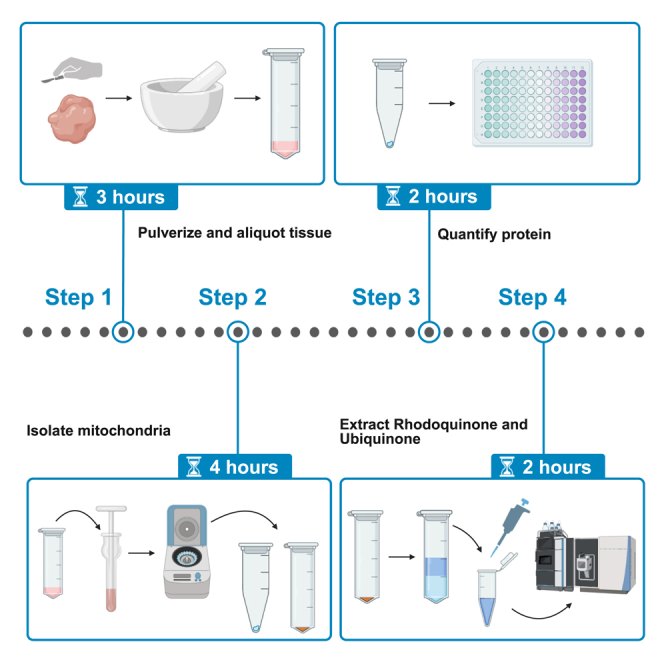

## Before you begin

### Background

The mitochondrial electron transport chain (ETC) serves multifaceted metabolic functions in cells.^2^^,^^3^ The mammalian ETC is a series of reduction and oxidation reactions whereby electrons are deposited on an electron carrier, transferred between ETC complexes, and terminally delivered to either O_2_ or fumarate.^4^^,^^5^ Ubiquinone (UQ) was previously thought to be the only quinone electron carrier in mammals, however, Valeros, *et al.* recently identified that certain mammalian tissues possess Rhodoquinone (RQ).^1^ RQ was previously known to be an electron carrier in bacteria^6^^,^^7^^,^^8^^,^^9^ and eukaryotes such as snails; mussels; oysters^10^; and nematodes,^11^^,^^12^ parasitic helminths,^13^^,^^14^ and protists.^15^ Similar to its roles in other organisms, RQ carries electrons to fumarate, instead of O_2_ in the mammalian ETC. As the flow of electrons between RQ and fumarate preserves mitochondrial function in an O_2_-independent manner, reprogramming the ETC to this pathway mitigates hypoxia-induced damage to mammalian cells and tissues.^1^

The physiological level of RQ in mammals is significantly lower than UQ, ranging between 1:5 and 1:100 across tissues, implying cell-type and/or regional specificity of this metabolite class. The low abundance of RQ necessitates mitochondrial purification for optimal detection. RQ is present in most mouse and human tissues, but is most abundant in the kidney, brain, and pancreas. Here, we present a comprehensive protocol to isolate mitochondria from tissues, extract RQ and UQ, and measure their levels using high resolution mass spectrometry. The protocol below describes the specific steps for isolating RQ from mouse tissues, however, this workflow can similarly be applied to RQ detection in human tissues, biofluids, and cultured cells with minor modifications.

### Before you begin

1. Label four sets of 1.5 mL microcentrifuge tubes.
2. Label two sets of 2 mL microcentrifuge tubes.
3. Prepare the mitochondria isolation buffer and pre-cool at 4° C before use.
4. Make a 1X diluted stock of Radioimmunoprecipitation Assay (RIPA) buffer and keep cool on ice. RIPA buffer should be stored at −20° C when not in use.
5. Prepare the extraction buffers (acidified LC-MS grade methanol and LC-MS grade hexane) and pre-cool at 4° C before use. Use glassware that is designated for LC-MS use only and has had no exposure to detergent (such as soap).

***Note:*** Detergents suppress ionization of metabolites in the source of the mass spectrometer. Any water used to clean LC-MS glassware should be ultrapure to avoid contamination of buffers that can cause ion suppression. Additionally, the detergent-based RIPA buffer should only be used for the aliquot of mitochondria designated for protein quantification and have no direct contact with the aliquot of mitochondria designated for LC-MS analysis.

6. For best results, make LC mobile phase solvents A and B right before use. Use glassware that has had no exposure to detergent.

### Institutional permissions

All mouse work was performed in accordance with UMass Chan Medical School IACUC policies. Experiments to extract UQ and RQ from mouse tissues will require individuals to acquire the appropriate permissions from their institution.

## Key resources table

**Table:** 

REAGENT or RESOURCE	SOURCE	IDENTIFIER
**Chemicals, peptides, and recombinant proteins**		

LC-MS grade hexane	Honeywell	Cat#34986-4X2.5L
LC-MS grade methanol	Fisher Scientific	Cat#A452SK-4
LC-MS grade acetonitrile	Sigma-Aldrich	Cat#34851-4L
LC-MS grade formic acid	Sigma-Aldrich	Cat#F0507-1L
LC-MS grade water	Sigma-Aldrich	Cat#270733-4X4L
Ammonium formate for HPLC, ≥99.0%	Honeywell	Cat#17843-50G
Hydrochloric acid, 37%, ACS reagent	Sigma-Aldrich	Cat#320331-500ML
Sucrose	Sigma-Aldrich	Cat#S0389
EGTA (ethyleneglycol- bis(β-aminoethyl)-N,N,N′,N′-tetraacetic Acid)	Sigma-Aldrich	Cat#324626-25GM
Tris base (Tris(hydroxymethyl)aminomethane)	Fisher Scientific	Cat#BP152-500
RIPA (radioimmunoprecipitation assay) lysis buffer, 10X	EMD Millipore	Cat#20-188

**Critical commercial assays**		

Pierce BCA protein assay kit	Thermo Fisher Scientific	Cat#23227

**Experimental models: Organisms/strains**		

Mouse: wild-type C57BL/6J, 8- to 16-week-old male and female	The Jackson Laboratory	RRID:IMSR_JAX:000664

**Software and algorithms**		

TraceFinder 5.1 SP1	Thermo Fisher Scientific	https://www.thermofisher.com/us/en/home/industrial/mass-spectrometry/liquid-chromatography-mass-spectrometry-lc-ms/lc-ms-software/lc-ms-data-acquisition-software/tracefinder-software.html

**Other**		

Mortar and pestle, porcelain	Eisco	Cat#CH0540B
Fisherbrand semimicro spatula	Fisher Scientific	Cat#14-374
VWR tissue grinders, PTFE, glass vessel	VWR	Cat#89026-386
VWR tissue grinders, PTFE, plain plunger	VWR	Cat#89026-398
Q Exactive Orbitrap mass spectrometer with an Ion Max source and HESI II probe	Thermo Fisher Scientific	N/A
Vanquish Horizon UHPLC system	Thermo Fisher Scientific	N/A
CentriVap benchtop vacuum concentrator	Labconco	N/A
CentriVap-105 cold trap	Labconco	N/A
Luna 3 μm PFP(2) 100 Å, LC column 100 × 2 mm	Phenomenex	Cat#00D-4447-BB0
Liquid nitrogen	N/A	N/A
Dry ice	N/A	N/A
Vortex-Genie 2	Scientific Industries	Cat#SI-0236
Vortex-Genie mixers, Scientific Industries, microtube foam insert	Scientific Industries	Cat#504-0234-00
Vortex-Genie 6-inch platform	VWR	Cat#58815-212
Micro centrifuge tube; sterile; 1.5 mL	Wilkem Scientific	Cat#72316005
9 mm screw cap for autosampler vial	Ibis Scientific	Cat#4400-32B
Milli-Q H_2_O	N/A	N/A
SureSTART 2 mL polypropylene screw top	Fisher Scientific	Cat#03452261

## Materials and equipment

**Table:** Mitochondria isolation buffer

Reagent	Final concentration	Amount
Sucrose	0.2 M	6.846 g
Tris Base	10 mM	0.1211 g
0.1 M EGTA/Tris	1 mM	1 mL
MilliQ H_2_O	N/A	Up to 99 mL
**Total**	**N/A**	**100 mL**

Adjust the pH to 7.4 using 1 M HEPES. Do NOT use NaOH to pH the buffer, as the sodium will damage mitochondria. Store at 4°C during active use. Store at −20°C if storing for more than two days. Maximum storage time to keep buffer integrity is one week.

**Table:** Acidified LC-MS grade methanol

Reagent	Final concentration	Amount
LC-MS-grade Methanol	99.9%	97.26 mL
1 M HCl	27.4 mM	2.74 mL
**Total**	**N/A**	**100 mL**

Store at 4°C wrapped in aluminum foil to protect from light. Can be stored at −20°C to pre-cool buffer before use. Maximum storage time is one month.

**CRITICAL:** Hydrogen chloride (HCl) is a corrosive agent that can cause harm to skin, eyes, and respiratory systems. Adhere to proper risk prevention measures during use. Measure in a ventilated fume hood.

**Table:** LC-MS resuspension buffer

Reagent	Final concentration	Amount
LC-MS-grade Methanol	N/A	200 mL
Ammonium formate	2 mM	0.0252 g
**Total**	**N/A**	**200 mL**

Store at 4°C wrapped in aluminum foil to protect from light. Can be stored at −20°C to pre-cool buffer before use. Maximum storage time is one month.

**CRITICAL:** Ammonium formate is an inhalation and corrosive hazard. Use caution and weigh inside a ventilated fume hood.

**Table:** LC mobile phase solvent A

Reagent	Final concentration	Amount
LC-MS-grade Water	N/A	999 mL
LC-MS-grade Formic Acid	0.1%	1 mL
**Total**	**N/A**	**1 L**

Store at room temperature. Maximum storage time is one month.

**CRITICAL:** Formic acid is an inhalation and corrosive hazard. Measure in a ventilated fume hood.

**Table:** LC mobile phase solvent B

Reagent	Final concentration	Amount
LC-MS-grade Acetonitrile	N/A	999 mL
LC-MS-grade Formic Acid	0.1%	1 mL
**Total**	**N/A**	**1 L**

Store at room temperature wrapped in aluminum foil to protect from light. Maximum storage time is one month.

**CRITICAL:** Acetonitrile and formic acid are inhalation hazards. Measure only in a ventilated fume hood.

## Step-by-step method details

### Harvesting and aliquoting tissue

**Timing: Approximately 3 h to pulverize and aliquot 20 samples**

***Note:*** This protocol describes the harvesting steps for mouse tissue, however this may also be performed on human tissue in a similar manner.

**CRITICAL:** After snap freezing the dissected tissues in liquid nitrogen, all tissues must be kept on dry ice, both before and after pulverizing, and stored at −80°C to prevent thawing.

This step ensures the stabilization of metabolites in the mouse tissues. Pulverizing by hand with a mortar and pestle creates a fine powder, which will be beneficial during mitochondrial purification.

***Note:*** Other methods for pulverizing the tissue may also be used to obtain similar results, such as cryogenic milling.

1. Dissect mouse tissues and place in a 1.5 mL microcentrifuge tube.
2. Immediately snap freeze tissues by submerging in a liquid nitrogen bath.

**CRITICAL:** Some microcentrifuge tubes will break open when exposed to the liquid nitrogen. We recommend testing your specific type of tube before use, or the catalog number is provided for the specific tube we use in this protocol.

3. Get a bucket of dry ice and place a porcelain mortar (we recommend a mortar about 10 cm in diameter) and pestle along with a metal spatula on the ice to become cold.

***Note:*** This will take a couple of minutes. You want the tools to be cold enough so that the powder remains frozen and does not clump together due to thawing.

4. Pour approximately 3 cm of liquid nitrogen into the mortar.

***Note:*** During pulverization, the liquid nitrogen will evaporate over time. Try to complete pulverizing before the nitrogen evaporates completely. Add additional nitrogen as needed.

5. Remove tissue from the microcentrifuge tube and place in the liquid nitrogen in the mortar.

***Note:*** Some tissues will adhere to the interior of the microcentrifuge tube; it is helpful to tap the tube upside down against a hard surface to loosen the tissue. The cold metal spatula may also be used to remove tissue from the tube. Careful placement of the tissue into the microcentrifuge tube prior to snap freezing may also alleviate this issue.

6. While the tissue is submerged in the liquid nitrogen, gently tap the tissue with the pestle until it is a fine powder.

***Note:*** If you run out of liquid nitrogen before the tissue is fully pulverized, you can add more to the mortar. However, be careful and pour slowly so that the tissue that is currently in the mortar is not ejected from the force.

**Figure 1:**
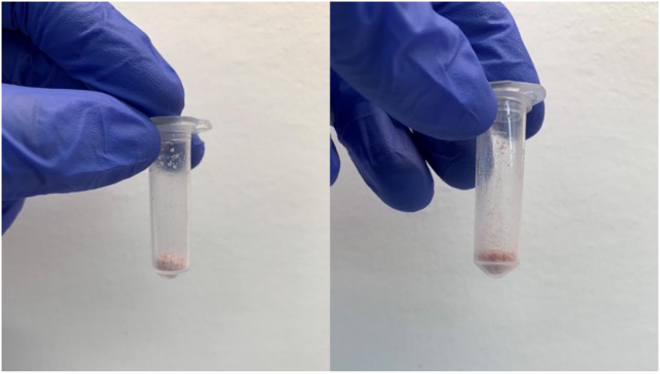
Example aliquots of tissue powder to be used for mitochondrial isolation The left image is a pulverized mouse brain and the right image is pulverized mouse kidney.

***Note:*** Slight variations in the amount of tissue aliquoted will not impact the results, as this protocol contains steps to normalize the data to the mitochondrial content extracted later from this aliquot. For best results in transferring and weighing tissue material, the tool you use to scoop the tissue powder should be kept cool and on dry ice along with the microcentrifuge tubes. This will prevent the tissue powder from warming up and sticking to the tool and/or sides of the tube.

7. Keeping the tubes on dry ice at all times, aliquot approximately 100 mg of tissue powder into a pre-cooled 2 mL microcentrifuge tube to be used for mitochondrial isolation (Figure 1). Use a cold metal spatula to scrape the tissue powder off the bottom of the mortar and into the tube.

8. Save the remaining tissue in the −80°C freezer for troubleshooting or other assays, such as polar metabolite profiling.
9. Clean the mortar, pestle, and any tool that touched the tissue by first wiping them clean with a dry paper towel and then rinsing repeatedly with deionized water. Dry thoroughly (do not leave any water behind) and return the supplies to dry ice to pulverize the next sample.

**Pause Point:** Store the aliquots of tissue powder for mitochondria isolation in the −80° C freezer until you are ready to proceed with the next step.

### Isolation of crude mitochondrial pellet from tissue

**Timing: Approximately 4 h/20 samples**

**CRITICAL:** All samples, tools, and buffers must be kept on regular ice. Do not use dry ice for this step as multiple freeze/thaw cycles on purified mitochondria can permeabilize them, causing metabolite leakage).

This step adapts a protocol to isolate mitochondria,^16^ to later be used for RQ extraction. Douncing in the homogenizer lyses the cells while keeping the mitochondria intact.

10. Cool mitochondrial isolation buffer on regular ice.
  a. If the buffer was being stored in the −20°C freezer, thaw the isolation buffer in a 37°C water bath.

**CRITICAL:** Don’t allow the isolation buffer to get too warm; thaw it only long enough to dissolve most of the ice. Once ice is no longer visible, shake to mix and place on regular ice.

**Figure 2:**
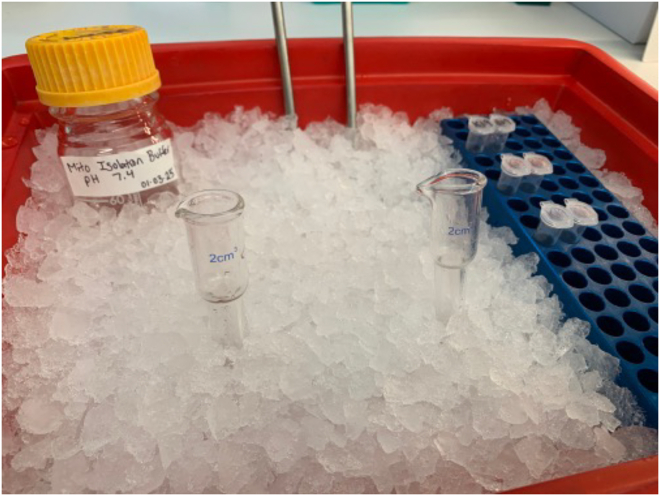
Mitochondrial isolation setup with homogenizers, isolation buffer, and tubes submerged in the ice bucket
12. Thaw the mouse tissue aliquots on the regular ice.

***Note:*** The mouse tissue will thaw quickly. This will only take 5-10 minutes.

11. Take a glass homogenizer and pestle and allow to cool on regular ice. Make sure the homogenizer is kept cold by burying the bottom portion in the ice (Figure 2).

13. Add 1 mL of isolation buffer to the tube with the aliquot of tissue powder.
  a. Invert the tube two to three times to get the tissue floating in the buffer.
  b. Pour directly into the homogenizer.

**CRITICAL:** Do not vortex any of the tubes in this section, as this could damage the mitochondria.

14. Add another 1 mL of isolation buffer to the 2 mL tube.
  a. Invert once again to get as much of the tissue as possible into the buffer.
  b. Pour into the homogenizer.
15. Leaving the homogenizer submerged in ice, slowly homogenize the solution by moving the pestle up and down for 30 strokes.

**CRITICAL:** Avoid creating air bubbles by slowly lifting the pestle up and down, being careful not to bring the pestle up past the top of the liquid. Excess bubbles will reduce the number of intact mitochondria at the end.

16. Pour the homogenate into a new 2 mL microcentrifuge tube.
17. Clean the homogenizer and pestle between each sample.
  a. Rinse both in deionized water, making sure there are no pieces of tissue left in or on the tools.

***Note:*** You may also flick the homogenizer upside down to disperse any excess deionized water so that it does not interfere with your samples.

***Note:*** Make sure the homogenizer is fully dried before moving on to the next sample.

18. Repeat steps 13–17 for each tissue sample.
19. Spin the tubes in a refrigerated 4 degrees C centrifuge at 600 x g for 10 minutes.

**Figure 3:**
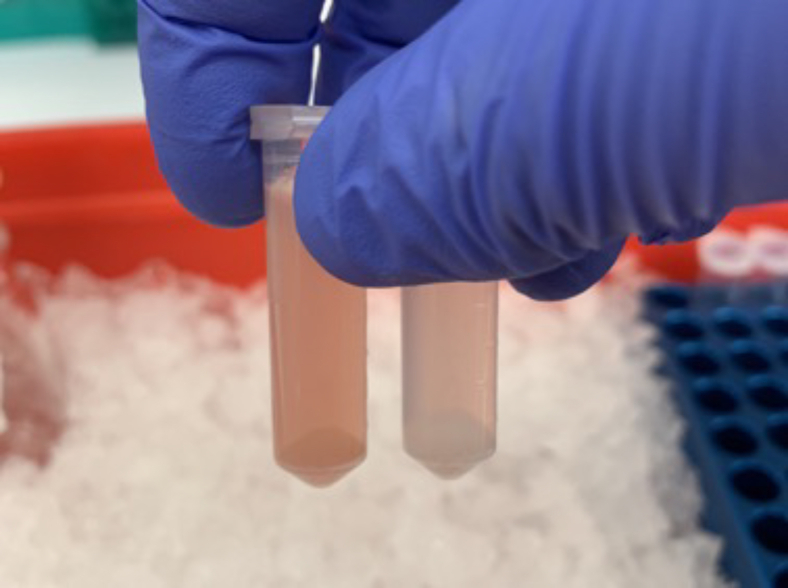
Pelleted cell debris and nuclei from homogenate after 600 x g centrifugation The left tube contains kidney and the right contains brain.

***Note:*** This step will pellet the cell debris and nuclei for removal. The supernatant should be cloudy (Figure 3). The supernatant has the mitochondria, which will be transferred to the next tube to be pelleted in the next step.

20. Use a p1000 pipette to transfer the supernatant (contains the mitochondria) 1 mL at a time into a new 2 mL microcentrifuge tube. Troubleshooting 1.

***Note:*** Be careful not to disturb the pellet. The tube with the cell debris pellet can now be discarded.

21. Spin the tubes containing the supernatant in a refrigerated 4 degrees C centrifuge at 7,000 x g for 10 minutes.

**Figure 4:**
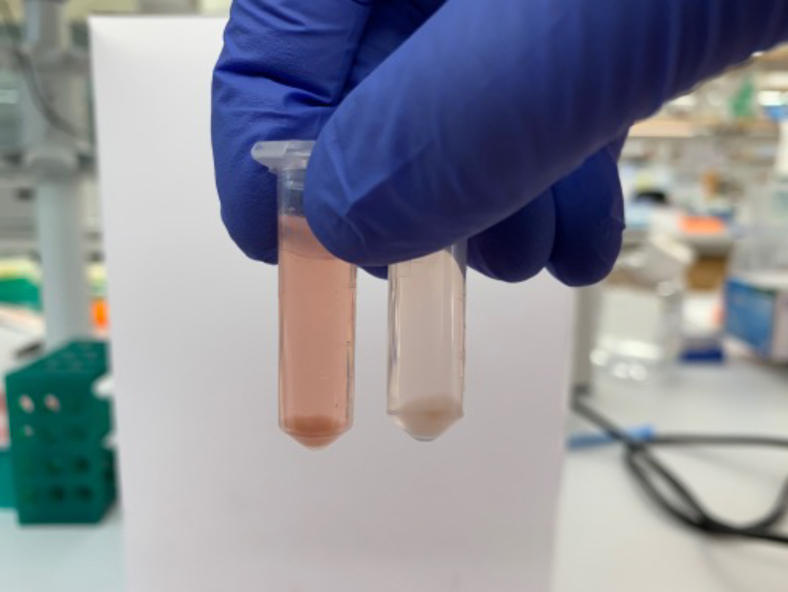
Crude mitochondria pellet after 7,000 x g centrifugation The left tube contains kidney and the right contains brain.

***Note:*** This step will pellet the mitochondria. The pellet should be brown in color depending on the amount of mitochondria in the tissue and the tissue subtype. Kidney have higher mitochondrial content and lower lipid content than brain, thus the lighter appearance of the pellet in the brain samples. (Figure 4).

22. Discard the supernatant, being sure not to disturb the pelleted mitochondria. Use a p1000 pipette and discard the supernatant in 1 mL increments.
23. Re-suspend the mitochondria in 2 mL of the isolation buffer by pipetting up and down.
24. Spin the tubes containing the mitochondria supernatant in a refrigerated 4 degrees C centrifuge at 7,000 x g for 10 minutes.
25. Discard the supernatant, being sure not to resuspend the pelleted mitochondria. Use a p1000 pipette and discard the supernatant 1 mL at a time.
26. Re-suspend the mitochondria in 1 mL of mitochondrial isolation buffer by pipetting up and down.
27. Transfer 25 μL of the re-suspended mitochondria into a new 1.5 mL microcentrifuge tube to be used for protein quantification.
  a. The remaining 975 μL will remain in the 2 mL tube.

***Note:*** The 1.5 mL tube contains an aliquot of the sample for protein quantification that will later be used to normalize the samples for equal loading into the mass spectrometer. The 2 mL tube with the remaining 975 μL of material will be pelleted and used for RQ extraction.

**Figure 5:**
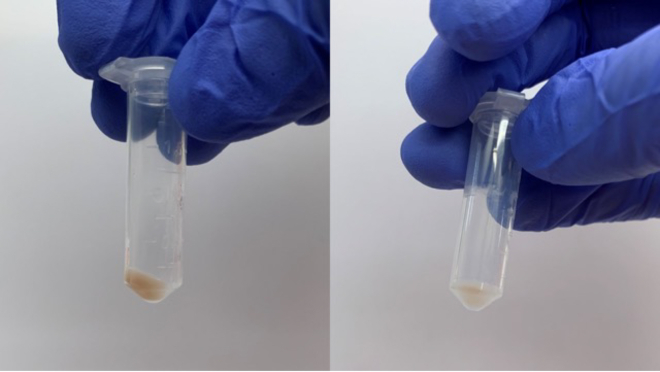
Examples of isolated, crude mitochondria pellets from kidney (left) and brain (right)
  b. Use a p1000 pipette and discard the supernatant 1 mL at a time. Troubleshooting 2.

28. Spin all the tubes in a refrigerated 4 degrees C centrifuge at 7,000 x g for 10 minutes.
29. Remove and discard the supernatant from all tubes.
  a. A brown, crude mitochondrial pellet should be left (Figure 5).

**CRITICAL:** Be sure not to disturb the pelleted mitochondria when removing the supernatant.

**Pause Point:** Store the pelleted mitochondria for protein quantification and RQ extraction in a −80°C freezer until ready to begin the metabolite or protein extraction steps.

### Quantifying protein from isolated mitochondria

**Timing: 2 h/20 samples**

Protein quantification is used to determine the amount of mitochondria in each sample. These values will be used to normalize input of material into the LC-MS prior to UQ and RQ analysis.

***Note:*** Below are steps to quantify protein using a BCA assay. This step should only be performed on the 25 μL aliquot of each sample. Although a BCA assay is described below, most protein quantification methods (including Bradford) will suffice for this step.

30. Remove the aliquots of mitochondria for protein quantification from the freezer and place on ice (not dry ice).
31. Add 250 μL of 1X RIPA buffer to the tube and pipette up and down.
32. Vortex samples for 10 minutes at 4°C in a cold room.
  a. If you do not have access to a cold room or a vortex that can hold microcentrifuge tubes, you may vortex each sample by hand as described below:
    i. Hold the sample on the vortex for a maximum of 2 minutes before returning to dry ice to keep the sample cold.
    ii. Keep the sample on dry ice for 1 minute.
    iii. Repeat these steps until you have vortexed each sample for a total of 10 minutes.
33. Spin the tubes in a refrigerated 4°C centrifuge at top speed (or at least 16,000 x g) for 10 minutes.
34. Quantify protein concentration using the Pierce BCA Protein Assay Kit. Troubleshooting 3.

***Note:*** It is recommended to do duplicates for both the standards and the samples in the 96-well plate.

35. Calculate how much resuspension buffer should be added to each sample to resuspend at an equal concentration of 20 μg/μL of protein. For example, if the protein quantification of the mitochondria comes out to be 1 μg/μL, do the following calculations:

Protein in the aliquot = 1 μg/μL x 250 μL RIPA buffer = 250 μg protein

μg/μL mitochondrial isolate = 250 μg protein /25 μL aliquot of mitochondria = 10 μg/μL.

Total protein in metabolite extraction aliquot = 10 μg/μL x 975 μL = 9750 μg.

Volume to resuspend final extract to achieve 20 μg/μL = 9750 μg/20 μg/μL = 487.5 μL.

### Non-polar metabolite extraction on crude mitochondrial pellets

**Timing: 2 h/20 samples**

This biphasic extraction separates the polar and non-polar metabolites to create a non-polar fraction that contains UQ and RQ. The methanol is acidified to preserve the redox status of the quinones.^16^ The non-polar fraction is dried down and concentrated using a refrigerated speedvac which will later be redissolved according to protein amounts determined in the previous step “quantifying protein from isolated mitochondria.”

36. Place the aliquot of mitochondria dedicated to UQ and RQ extraction in a bucket of dry ice.
37. Place the LC-MS grade acidified methanol and hexane on dry ice and allow to cool.
38. Add 500 μL of LC-MS grade acidified methanol to each tube.
39. Vortex samples for 15 minutes at 4°C in a cold room.
40. Bring the samples on dry ice into a chemical fume hood.
41. Add 500 μL of LC-MS grade hexane to each tube inside the fume hood.
42. Vortex samples for 15 minutes at 4°C in a cold room.
43. Spin the tubes in a refrigerated 4°C centrifuge at 21,300 x g (or top speed) for 10 minutes.

***Note:*** This will fully separate the polar and non-polar layers. The top layer is the hexane layer containing the non-polar metabolites, including RQ and UQ. Other non-polar extraction buffers such as chloroform have not worked well in our hands for UQ and RQ extraction. The bottom layer is the methanol layer and there may be a visible debris layer pelleted at the very bottom of the tube.

44. Using a p1000 pipette, transfer the top layer into a new 1.5 mL microcentrifuge tube. The rest of the tube and its contents may now be discarded.
45. Dry the hexane layer down in a refrigerated speedvac. The centrivap was operated in a range of −4°C to 4°C and the cold trap set to −105°C.

***Note:*** If you do not have access to a refrigerated speedvac, the samples can alternatively be dried down using a nitrogen evaporator.

**Pause Point:** If you are not able to dry down the tubes immediately after extraction, you may store them in the −80°C freezer.

***Note:*** The drying down process for a hexane layer can be anywhere from 1–4 hours depending on the efficiency of your speedvac. Samples should all be dried down at the same time and in one continuous session (without partially drying down, storing, and drying down the rest later). The sample is considered fully dried down when there is no liquid left in the tube and there is a visible metabolite pellet.

### Resuspension and preparation for analysis on the LC-MS

**Timing: 1 h/20 samples**

***Note:*** Label one set of autosampler vials and keep covered with aluminum foil until they are filled and capped to prevent any dust from settling in the vials.

This step prepares the samples to be run on an LC-MS method to detect and measure non-polar metabolites. Keeping the samples in an autosampler held at 4 degrees C prevents the degradation of the metabolites during the run.

46. Place the dried down samples on ice (not dry ice).
47. Add the appropriate amount of the LC-MS resuspension buffer to achieve 20 μg/μL based on the protein quantifications done in step 35.
48. Vortex the samples for 10 minutes at 4 degrees C in a cold room.
49. Pipette 20 μL of each sample into an autosampler vial.

**CRITICAL:** The volume to aliquot into autosampler vials can vary depending on injection needle offset parameters of the LC-MS setup. We recommend putting at least 20 μL volume in each vial, but higher volumes will also work. Be sure there are no bubbles in the autosampler vial to avoid injecting air instead of sample.

50. The samples should be kept on regular ice while you set up the LC-MS for the run.
51. It is recommended if you are running different tissues in the same run, to put at least one extraction solvent blank in between them. It is preferable to run the tissues in batches.
52. A typical LC-MS run will begin with 2-3 blanks (i.e. the LC-MS resuspension buffer) and a mock injection of your first sample to condition the column before the run.
53. Then the samples can be queued either grouped together by tissue type with an extraction solvent blank between tissue subtypes, or ran in random order with a blank between each sample.
54. If desired, quality control pools and/or rhodoquinone standards can be queued after the samples.
55. 2-3 blanks should be included at the end of the LC-MS run.

***Note:*** Each tissue will have a different matrix (some tissues have more lipids than others) which can impact the sensitivity and linear range of detection on the LC-MS.

56. The column is the Luna 3 μm PFP(2) 100 Å, LC Column 100 x 2 mm from Phenomenex.
57. Prepare LC Mobile Phase Solvent A and LC Mobile Phase Solvent B.
58. Set the gradient of the column to be as below:

**Table:** LC gradient

Mobile phase solvent B %	Time (minutes)
Hold at 70%	3 min
Increase to 98%	0.25 min
Hold at 98%	1.75 min
Increase to 99%	1 min
Hold at 99%	2.75 min
Decrease to 70%	1.75 min
Hold at 70%	2 min

59. Run at a flow rate of 0.5 mL/min.

***Note:*** Other reversed phase chromatography methods have successfully been used to detect RQ in mammals, for example human plasma run on a C18 chromatography.^17^

60. Operate the mass spectrometer with the following parameters:

**Table:** Mass spectrometer parameters

Parameter	Setting
MS acquisition mode	Full scan
m/z acquisition range	600–1000 m/z
Ionization mode	Positive
Spray voltage	4.0 kV
Heated capillary temperature	320°C
HESI probe temperature	30°C
Sheath gas flow	40 units, room temperature
Auxiliary gas	15 units, room temperature
Sweep gas flow	1 unit, room temperature
Scan resolution	17,500
AGC target	3 x 10^6^
Maximum injection time	250 ms

61. If necessary, include an additional scan in the select ion monitoring (SIM) mode alongside the full scan to enhance RQ detection.
  a. As the abundance of RQ is lower than UQ, some tissues require a SIM method to increase sensitivity.
  b. The neutral molecular formula of RQ_9_ (mouse tissues) is C_53_H_81_NO_3_ and RQ_10_ (human tissues) is C_58_H_89_NO_3_. The proton and sodium adducts are dominant in the tissue samples measured, although these adducts can change depending on the tissue sample, glassware used, and extraction method. Below are the exact masses for each adduct.
    **Table:** RQ SIM masses

Adduct	Mass (m/z)
RQ-9 [H^+^]	780.62892
RQ-9 [Na^+^]	802.61087
RQH_2_-9 [H^+^]	782.64457
RQH_2_-9 [Na^+^]	804.62652
RQ-10 [H^+^]	848.69152
RQ-10 [Na^+^]	870.67347
RQH_2_-10 [H^+^]	850.70717
RQH2-9 [Na+]	872.68912
  c. Set up the SIM method with the following parameters:

**Table:** SIM method parameters

Parameter	Setting
MS acquisition mode	SIM mode
Ionization mode	Positive
Spray voltage	4.0 kV
Heated capillary temperature	320°C
HESI probe temperature	30°C
Sheath gas flow	40 units, room temperature
Auxiliary gas	15 units, room temperature
Sweep gas flow	1 unit, room temperature
Scan resolution	17,500
AGC target	3 x 10^6^
Maximum injection time	250 ms

62. Inject 2 μL of each sample on the LC-MS.
63. Once the LC-MS run is completed, analyze the data in Tracefinder 5.1 SP1 (Thermo Fisher Scientific).
64. To confirm the detection of UQ and RQ, MS/MS (fragmentation) analysis should be performed using a product reaction monitoring (PRM) method.
  a. Set up the MS/MS method with the following parameters:

***Note:*** The monitoring window will depend on the chromatography used.

**Table:** MS/MS method parameters

Parameter	Setting
MS acquisition mode	PRM mode
Ionization mode	Positive
Spray voltage	4.0 kV
Heated capillary temperature	320°C
HESI probe temperature	30°C
Sheath gas flow	40 units, room temperature
Auxiliary gas	15 units, room temperature
Sweep gas flow	1 unit, room temperature
Scan resolution	17,500
AGC target	2 x 10^5^
Maximum injection time	250 ms
Isolation window	0.5 m/z
Stepped NCE	30 units

**Table:** UQ and RQ PRM masses

Adduct	Mass (m/z)	Monitoring window
RQ-9 [H^+^]	780.62892	4–6 minutes
RQH_2_-9 [H^+^]	782.64457	4–6 minutes
UQ-9 [H^+^]	795.62859	5–7 minutes
UQH_2_-9 [H^+^]	797.64424	5–7 minutes

65. Inject 2 μL of each sample on the LC-MS.
66. Once the LC-MS run is completed, analyze the data in Tracefinder 5.1 SP1 (Thermo Fisher Scientific).

## Expected outcomes

As mitochondria are brown in color, you should expect to see a sizable brown mitochondria pellet after the crude mitochondrial isolation steps are complete (Figure 5). In the final step before loading samples on the LC-MS, the expected resuspension values to achieve a 20 μg/μL final concentration should be around 20-500 μL for the estimated 100 mg of tissue starting material. Replicates of the same tissue should be around the same range of total protein amount and thus will have similar resuspension values. A tissue with a high amount of mitochondria such as the liver will have higher resuspension values than a tissue with lower mitochondrial content such as the white adipose tissue.

**Figure 6:**
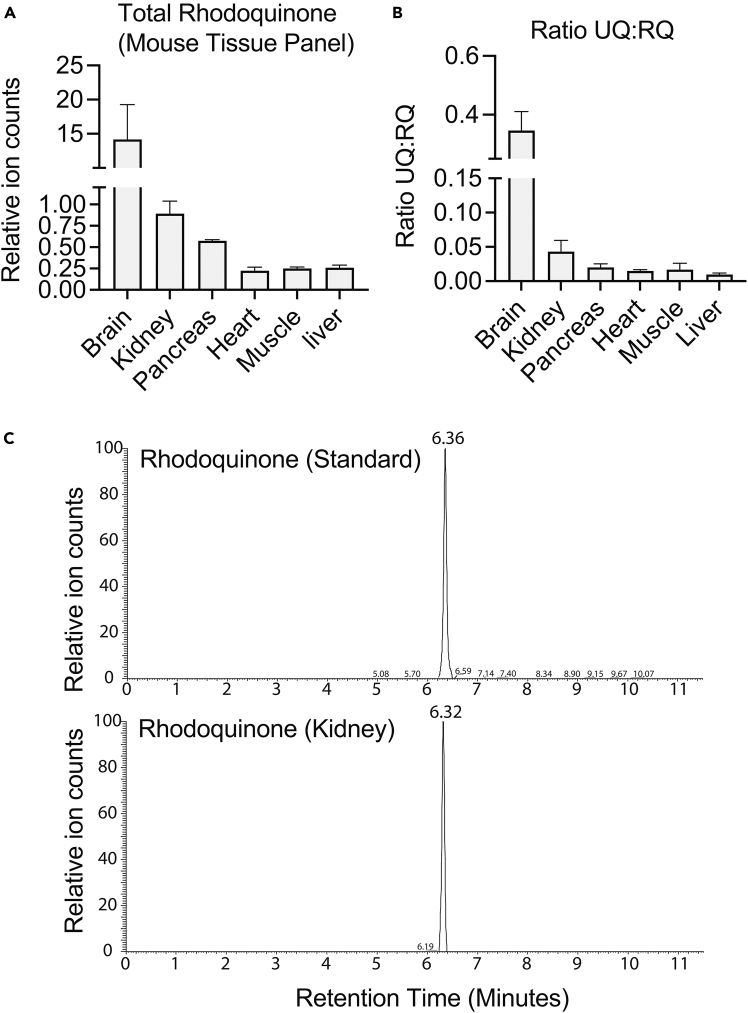
Sample rhodoquinone detection in mouse tissues (A) Total rhodoquinone in mouse brain, kidney, pancreas, heart, muscle, and liver. Data represent mean +/− SEM. (B) Ratio of ubiquinone (UQ) to rhodoquinone (RQ) in mouse brain, kidney, pancreas, heart, muscle, and liver. Data represent mean +/− SEM. (C) Chromatogram of a rhodoquinone standard and rhodoquinone detected in mouse kidney showing the same retention time.

RQ detection is tissue-specific with the highest amounts in the brain, kidney, and pancreas (Figure 6A). The levels of RQ: UQ in most tissues will be approximately 1:100 (Figure 6B). The retention time, exact mass, and fragmentation patterns of rhodoquinone in mouse tissues should match that of purified standards (Figure 6C). The method to purify RQ standards can be found in the materials and methods section of Valeros *et al*.^1^

## Quantification and statistical analysis

We recommend that LC-MS data is analyzed using Tracefinder 5.1 SP1 (Thermo Fisher Scientific). Peaks should be integrated according to a stringent 5 ppm mass tolerance and minimal shifts in retention time compared to standards. The area under the curve for all adduct integrations should be summed together to get a total abundance of RQ and UQ, respectively. The peak areas for UQ and RQ can be integrated simultaneously on tissues processed together on the LC-MS. These values do not need to be normalized because samples should be loaded into the LC-MS with even protein quantifications. The ion counts of ubiquinone and rhodoquinone cannot be compared unless standard curves for each of these metabolites have been run and absolute quantification is performed.

## Limitations

Rhodoquinone is most enriched in mouse kidney, brain, and pancreas.^1^ Detection of RQ in other tissues may be challenging due to its low abundance. Moreover, some tissues such as the adipose tissue and brain have higher lipid content than others, making RQ detection more challenging in these models because of lipid contamination on the crude mitochondrial pellet. For studies comparing RQ levels in two model systems, it is important that mice are age and sex-matched, as the impact of these factors on RQ has not been determined yet.

## Troubleshooting

### Problem 1

Excessive lipids in the tissue homogenate after 600 x g centrifugation (related to step 20).

Tissues (i.e. the brain, adipose tissue) can have floating fat and lipids in the homogenate. This lipid content will not get pelleted in the initial 600 x g centrifugation step, but you want to avoid pelleting it with the mitochondria at the later, higher speed centrifugation step. Excessive lipids will interfere with the mitochondrial pellet and can later suppress the signal on the LC-MS analysis of a sample.

### Potential solution

• Avoid the lipid layer with careful pipetting: The lipids can present as either a floating mass at the top of the supernatant or they may be stuck to the sides of the tube. Insert your pipette tip as far away from the lipids as possible and pipette up the supernatant slowly. Pipetting smaller amounts of the supernatant at a time can also decrease your chance of lipid contamination.
• Aspirate the lipid layer: In the extreme case that your sample has a thick lipid layer at the top, using a thin aspirating pipet to remove it may be necessary. You may slowly aspirate away the lipid layer, being sure not to remove any of the homogenate supernatant.

### Problem 2

Lack of brown pellet after the isolation of crude mitochondria (related to step 29).

You should see a large, brown pellet of mitochondria after the isolation is complete. However, some tissues have low levels of mitochondria. When isolating mitochondria from tissues with lower amounts of mitochondria, it is common to see a lighter color pellet or no visible pellet, which will be problematic for RQ detection in later steps.

### Potential solution

• Increase amount of tissue: For tissues that have lower amounts of mitochondria, you may increase the amount of tissue you use in the isolation step.
• Test alternative homogenization methods: To increase the yield of mitochondria from a given tissue, you can increase or decrease the number of strokes during homogenization. Alternatively, one could utilize syringe lysis methods for mitochondrial isolation described here.^17^

### Problem 3

Inaccurate BCA results due to fat and lipids in the protein sample (related to step 34).

A cloudy protein extract can indicate there is too much lipid content in the sample which will interfere with a plate readers’ absorbance readings in the BCA assay.

### Potential solution

• Dilute sample: Diluting your protein sample in 1X PBS can reduce the effect that the lipid composition of a sample will have on the absorbance reading. The total volume of the BCA sample should be 25 μL and we recommend doing a 1:4 dilution of sample for best results.
• Use an alternative protein quantification method such as Bradford Assay.

### Problem 4

Low rhodoquinone detection in purified mitochondria (related to step 59).

### Potential solution

• Low amount of material due to inaccurate protein quantification: To address this, increase the amount of material injected into the LC-MS, up to 100 μg/μL (as opposed to the suggested 20 μg/μL).
• Low signal could be caused by lysed mitochondria during mitochondrial isolation. To address this, prepare mitochondria fresh and adjust the number of strokes during homogenization, periodically monitoring the sample under a light microscope to determine the minimum number of strokes required to lyse cells as described in this protocol.^18^
• Low signal can be due to inefficient extraction of lipids from the mitochondrial pellet or inefficient redissolution of the dried down metabolite extracts prior to LC-MS analysis. If you encounter difficulty resuspending the pellets using the suggested volumes of buffers, scale the buffers up until the pellets fully go into solution.
• Low detection could be due to low RQ levels in the tissue of interest. To address this, run the samples using the select ion monitoring (SIM) method to increase the sensitivity for RQ.
• Low signal can be caused by the ionization source on the mass spectrometer being dirty, which interferes with the ionization of metabolites in the samples. To improve sensitivity, clean the entire source housing, needle, sweep cone, and ion transfer tubes in a sonicating water bath submerged in 80:20 LC-MS grade methanol: formic acid for 15 minutes, followed by sonicating the components in 100% LC-MS grade methanol and allowing to fully dry.

## Resource availability

### Lead contact

Further information and requests for resources and reagents should be directed to and will be fulfilled by the lead contact, Jessica Spinelli (jessica.spinelli@umassmed.edu).

### Technical contact

Further information needed to carry out the techniques described in this protocol should be directed to the lead contact, Jessica Spinelli (jessica.spinelli@umassmed.edu).

### Materials availability

Reagents used in this study can be provided upon request through the lead contact. All other data are available in the main text.

### Data and code availability

• Metabolomics data have been deposited on Metabolomics Workbench. The project number is PR001988 and results are publicly available as of the date of publication (https://doi.org/10.21228/M8RJ07).
• This paper does not report original code.
• Additional information required to reanalyze the data reported in this paper is available from the lead contact upon request.

## Acknowledgments

This work was generously funded by the Worcester Foundation for Biomedical Research, the Smith Family Awards Program for Excellence in Biomedical Research, the Searle Scholars Program, AFAR Grant for Junior Faculty, the MassVentures Acorn Innovation Grant, the Pilot Project Grant from Breakthrough T1D, and the NIDDK Innovative Science Accelerator Program (36350-27) granted to J.B.S.

## Author contributions

M.J. performed all experiments and optimizations needed for the protocol. J.B.S. acquired funding for the study. M.J. and J.B.S. wrote the manuscript.

## Declaration of interests

J.B.S. is a co-inventor on a patent filed by the Whitehead Institute and UMass Chan Medical School on the uses of rhodoquinone for treatment of disease. J.B.S. is a co-inventor on a patent filed by the UMass Chan Medical School and Princeton University on the composition and usage of compounds for rhodoquinone-ETC-related diseases.
